# Interleukin-6 polymorphisms and risk of coronary artery diseases in a Chinese population: A case-control study

**DOI:** 10.12669/pjms.324.9908

**Published:** 2016

**Authors:** Yao Hongmei, Jia Yongping, Lv Jiyuan

**Affiliations:** 1 Yao Hongmei, Department of Cardiology, The First Hospital of Shanxi Medical University, No. 85 Xinjiannan Road, Taiyuan 030001, Shanxi, China; 2 Jia Yongping, Department of Cardiology, The First Hospital of Shanxi Medical University, No. 85 Xinjiannan Road, Taiyuan 030001, Shanxi, China; 3 Lv Jiyuan, Department of Cardiology, The First Hospital of Shanxi Medical University, No. 85 Xinjiannan Road, Taiyuan 030001, Shanxi, China

**Keywords:** Interleukin-6, -174G>C, -572G>C, -597G>A, Polymorphism, Coronary artery disease

## Abstract

**Objective:**

We aimed to evaluate the relationship between IL-6-174G>C, -572G>C and -597G>A polymorphisms and development of coronary artery diseases in a Chinese population.

**Methods:**

A total of 275 patients with coronary artery disease and 296 healthy control subjects were collected between January 2013 and November 2014. The IL-6 genotyping for -174G>C, -592G>C and -597G>A polymorphic sites was determined by polymerase chain reaction-restriction fragment length polymorphism (PCR-RFLP) according to the manufacturer instructions.

**Results:**

Using unconditional regression analysis, we observed that the AC and CC genotypes of IL-6 -592A>C were associated with the increased risk of developing coronary artery disease when compared with the AA genotype, and the adjusted ORs (95%CI) were 1.63(1.12-2.38) and 2.70(1.57-4.67), respectively. Additionally, the C allele of IL-6 -592A>C (OR=1.65, 95%CI=1.29-2.11) was correlated with a higher risk of developing coronary artery disease in comparison to the A allele. However, no relationship was found between IL-6-174G>C and -597G>A polymorphisms and coronary artery disease susceptibility.

**Conclusion:**

This study suggests that IL-6 -592G>C polymorphism is correlated with the risk of coronary artery disease. More well-designed prospective studies based on large sample size, multiple SNPs or haplotypes are required to confirm the current findings.

## INTRODUCTION

Coronary artery disease, namely coronary atherosclerotic heart disease, has become a challenging problem which severely threatens public health. Coronary artery disease is related with high morbidity and mortality worldwide, and the number of individuals in China suffering from CAD shows a tendency to increase.^1^ Even though improvements in early detection have reduced the mortality rates of coronary artery disease in recent years, prevention of coronary artery disease is always a main public health concern.

The pathogenesis of coronary artery disease occurs over a long period of time, involves many environmental and lifestyle factors, including hypertension, hyperlipaemia, diabetes, tobacco smoking, obesity and family history of atherosclerosis.^2^,^3^ Additionally, hereditary factors play an important role in the risk of coronary artery disease susceptibility, and previous published studies have revealed that many genetic factors play an important role in the development of this diseases, such as B-cell lymphoma 2 2, aldehyde dehydrogenase 2, cholesteryl ester transfer protein, interleukin-10 (*IL-10*) and scavenger receptor class B type 1.^4^-^9^

Molecular mechanisms underlying inflammation-correlated diseases included DNA damage, disruption of the immune response and alternation of normal cell microenvironment, which were all closely related with disequilibrium of inflammatory cytokines. Interleukin-6 (*IL-6*) is such a multifunctional pro-inflammatory cytokine produced by activated T cells, B cells, monocytes as well as cancerous cells. Since single nucleotide polymorphisms of *IL-6* gene promoter may affect the expression and secretion of *IL-6*, and subsequently the altered circulating levels might result in relevant biological responses, the *IL-6* polymorphism has been regarded as a crucial modulator in pathogenesis of various diseases, such as ischemic stroke, osteoporosis, dyslipidemia, hypertension and cardiovascular diseases.^10^-^14^

Currently, although several studies have investigated the correlation between *IL-6* polymorphisms and development of cardiovascular disease, but the conclusions are conflicting.^15^-^19^ We carried out a study to evaluate the relationship between *IL-6*-174G>C, -572G>C and -597G>A polymorphisms and development of coronary artery disease in a Chinese population.

## METHODS

This is a case-control study conducted between January 2013 and November 2014. Two hundred seventy five patients with coronary artery disease were enrolled from the First Hospital of Shanxi Medical University. All the new patients with coronary artery diseases were diagnosed using coronary angiography. The diagnosis of coronary artery disease was as follows: luminal stenosis above 50% in one of the main coronary arteries (left main coronary stenosis, anterior descending branch, circumflex coronary artery and right coronary artery) or their branch retinal arteries, and exhibiting stable angina, unstable angina pectoris and acute myocardial infarction. Patients who had a history of percutancous coronary intervention or coronary artery bypass graft intervention, congenital heart disease, rheumatic valvular heart disease, cardiomyopathy, severe heart failure, end-stage renal and liver disease, serious infections, malignant tumor, thyroid disease, connective tissue disease or immune system diseases were excluded

A total of 296 healthy control subjects were also enrolled from individuals who visited outpatient clinics at the First Hospital of Shanxi Medical University during the same time period. The control subjects were confirmed to be free of coronary artery diseases and other cardiovascular diseases, end-stage renal and liver disease, serious infections, malignant tumor, thyroid disease and immune system diseases.

The baseline variables of coronary artery disease patients and control subjects, including age, gender, tobacco smoking, alcohol consumption and body mass index (BMI) as well as family history of coronary artery diseases, were collected with a self-designed questionnaire. The clinical data of coronary artery disease patients and control subjects, including hypertension, type 2 diabetes mellitus, total cholesterol (TC), triglyceride (TG), low-density lipoprotein cholesterol (LDL-c) as well as high-density lipoprotein cholesterol (HDL-c), were collected from medical records. The study was carried out with the permission of the ethics committee of the First Hospital of Shanxi Medical University. A written informed consent was obtained from all the subjects before enrollment into this study. The ethical standards adopted for this study were in agreement with the standards of the Declaration of Helsinki.

### DNA extraction and genotyping

Five blood samples were collected from each investigated subject. The peripheral blood was kept in tube with EDTA and stored at -20°C until using. The extraction of the genomic DNA was done from the peripheral blood using QIAamp DNA Blood Mini Kit (QIAGEN, USA) following the manufacturer’s recommendation. The *IL-6* genotyping for -174G>C, -592G>C and -597G>A polymorphic sites was determined by polymerase chain reaction-restriction fragment length polymorphism (PCR-RFLP) according to the manufacturer instructions. The primers, restriction enzymes and PCR digested fragments shown in Table-I.

**Table-I:** Primers, restriction enzymes and PCR digested fragments of *IL-6*-174G>C, -572G>C and -597G>A

IL-6	Primers(5’-3’)	Restriction enzymes	Digested fragments
-174G>C	TGACTTCAGCTTTACTCTTTGT CTGATTGGAAACCTTATTAAG	NlaIII	GG: 164bp and 59bp GC: 164bp, 111bp, 59bp and 53bp CC: 111bp, 59bp and 53bp
-572G>C	GAGACGCCTTGAAGTAACTG AACCAAAGATGTTCTGAACTGA	BsrBI	GG: 122bp and 60bp GC: 182bp, 122bp and 60bp
-592G>A	CTCCTCTAAGTGGGCTGAAG CAAGCCTGGGATTATGAAGA	RsaI	GG: 412bp GA:412bp, 236bp and 176bp AA: 236bp and 176bp

PCR was performed in a 25 μl reaction mixture containing 10.5μl ddH_2_O, 12.5μl PCR MIX, 0.375μl of each primer (10pmol/μl), 0.25μl Taq enzyme (5U/μl) and 1μl DNA template (50ng/μl). The PCR condition was set at initial denaturation at 94°C for 3 minutes, followed by 35 cycles of denaturation at 94°C for 30 seconds, annealing at 56°C for 30 seconds, extension at 72°C for 60 seconds, and a final elongation of 5 mins at 72°C. Qualitative and quantitative estimation of DNA was carried out with agarose gel electrophoresis and spectrophotometric estimation.

### Statistical analysis

The differences within demographic, lifestyle and genotype frequencies between the two groups were analyzed using chi-square tests (χ^2^test) or student *t* test. The correlation between *IL-6*-174G>C, -572G>C and -597G>A genotype polymorphisms and susceptibility to coronary artery disease was evaluated using unconditional logistic regression analysis. Whether the *IL-6*-174G>C, -572G>C and -597G>A genotype frequencies were in agreement with the Hardy-Weinberg equilibrium was evaluated using chi-square tests. The results were estimated using Odds ratio (ORs) along with 95% Confidence Intervals (CI), and the results were adjusted for possible confounder. All the statistical analysis was done using IBM SPSS Statistics for Windows, Version 20.0. (Armonk, NY: IBM Corp).*P*-value <0.05 was regarded as statistically significant.

## RESULTS

The demographic, lifestyle and clinical characteristics of the study participants are presented in Table-II. By comparison with the control subjects, coronary artery disease patients were more likely to have higher age (t=1.78, P=0.04), higher BMI (t=3.09, P=0.001), have a family history of coronary artery disease (χ^2^=5.45, P=0.02), suffer from hypertension (χ^2^=20.16, P<0.001) and type 2 diabetes mellitus (χ^2^=10.01, P<0.001), and have higher level of TC (t=5.39, P<0.001) and LDL-c (t=4.31, P<0.001) as well as lower level of HDL-c (t=9.38, P<0.001). The controls and patients were comparable in respect to gender (χ^2^=2.45, P=0.12), tobacco smoking (χ^2^=0.18, P=0.67), alcohol consumption (χ^2^=0.08, P=0.77) and TG (t=1.35, P=0.09) level.

**Table-II:** The demographic, lifestyle and clinical characteristics of study subjects.

Variables	Patients n=275	%	Controls n=296	%	χ^2^ test or t test	P value
Age, years	62.64±8.43		61.43±7.85		1.78	0.04
Gender						
Females	86	31.27	111	37.50		
Males	189	68.73	185	62.50	2.45	0.12
BMI, kg/m^2^	26.41±2.56		25.75±2.54		3.09	0.001
Tobacco smoking						
No	92	33.45	104	35.14		
Yes	183	66.55	192	64.86	0.18	0.67
Alcohol consumption						
No	87	31.64	97	32.77		
Yes	188	68.36	199	67.23	0.08	0.77
Family history of coronary artery disease						
No	230	83.64	267	90.20		
Yes	45	16.36	29	9.80	5.45	0.02
Hypertension						
No	147	53.45	212	71.62		
Yes	128	46.55	84	28.38	20.16	<0.001
Type 2 diabetes mellitus						
No	205	74.55	252	85.14		
Yes	70	25.45	44	14.86	10.01	0.002
TC, mmol/L	4.64±1.15		4.08±1.32		5.39	<0.001
TG, mmol/L	1.52±1.19		1.64±0.93		1.35	0.09
LDL-c, mmol/L	2.84±0.95		2.48±1.04		4.31	<0.001
HDL-c, mmol/L	1.23±0.26		1.46±0.32		9.38	<0.001

BMI: body mass index; TC: total cholesterol; TG: triglyceride; LDL-c: low-density lipoprotein cholesterol; HDL-c: high-density lipoprotein cholesterol.

The genotype distributions of *IL-6*-174G>C, -592G>C and -597G>A are shown in Table-III. The genotype distributions of *IL-6*-174G>C (χ^2^=1.24, P=0.27) and -592G>C (χ^2^=2.40, P=0.12) were comparable between coronary artery disease patients and control subjects, whereas a significant difference was observed between coronary artery disease patients and control subjects in respect to *IL-6* -592G>C (χ^2^=15.87, P<0.001) (Table-III). Using chi-square test, we observed that the genotype distributions of *IL-6*-174G>C, -592G>C and -597G>A were in agreement with the HWE in both coronary artery disease patients (P values were 0.55, 0.79 and 0.71 for *IL-6*-174G>C, -592G>C and -597G>A, respectively) and control subjects (P values were 0.68, 0.89 and 0.51 for *IL-6*-174G>C, -592G>C and -597G>A, respectively).

**Table-III:** Genotype frequencies of *IL-6*-174G>C, -592G>C and -597G>A in the two study groups

Variables IL-6	Patients n=275	%	Controls n=296	%	χ^2^ test	P value	P for HWE	
							Patients	Controls
-174G>C								
GG	256	93.09	282	95.27				
GC	19	6.91	14	4.73				
CC	0	0.00	0	0.00	1.24	0.27	0.55	0.68
-592A>C								
AA	87	31.64	135	45.61				
AC	134	48.73	129	43.58				
CC	55	20.00	32	10.81	15.87	<0.001	0.79	0.89
-597G>A								
GG	263	95.64	274	92.57				
GA	12	4.36	22	7.43				
AA	0	0.00	0	0.00	2.40	0.12	0.71	0.51

HWE: Hardy-Weinberg equilibrium.

The relationship between *IL-6*-174G>C, -592G>C and -597G>A polymorphisms and development of coronary artery diseases is described in Table-IV. Using unconditional regression analysis, we observed that the AC and CC genotypes of *IL-6* -592A>C were associated with the increased risk of developing coronary artery disease when compared with the AA genotype, and the adjusted ORs (95%CI) were 1.63(1.12-2.38) and 2.70(1.57-4.67), respectively. Additionally, the C allele of *IL-6* -592A>C (OR=1.65, 95%CI=1.29-2.11) was correlated with an elevated risk of developing coronary artery disease in comparison to the A allele. However, no relationship was found between *IL-6*-174G>C and -597G>A polymorphisms and coronary artery disease susceptibility.

**Table-IV:** Relationship between IL-6-174G>C, -592G>C and -597G>A polymorphisms and development of coronary artery disease

Variables	Patients n=275	%	Controls n=296	%	OR(95%CI)^1^	P value
IL-6-174G>C						
GG	256	93.09	282	95.27	1.0 (Ref.)	-
GC	19	6.91	14	4.73	1.49 (0.69-3.29)	0.26
CC	0	0.00	0	0.00	-	-
Allele						
G	531	96.55	578	97.64	1.0 (Ref.)	-
C	19	3.46	14	2.37	1.48(0.69-3.22)	0.27
-592A>C						
AA	86	31.27	135	45.61	1.0 (Ref.)	-
AC	134	48.73	129	43.58	1.63(1.12-2.38)	0.01
CC	55	20.00	32	10.81	2.70(1.57-4.67)	<0.001
Allele						
A	306	55.64	399	67.40	1.0 (Ref.)	-
C	244	44.36	193	32.60	1.65(1.29-2.11)	<0.001
-597G>A						
GG	263	95.64	274	92.57	1.0 (Ref.)	-
GA	12	4.36	22	7.43	0.57(0.25-1.23)	0.12
AA	0	0.00	0	0.00	-	-
Allele						
G	538	97.82	570	96.28	1.0 (Ref.)	-
A	12	2.18	22	3.72	0.58(0.26-1.23)	0.13

1 Ajusted for age, gender, BMI, family history of coronary artery disease, hypertension, type 2 diabetes mellitus, TC, LDL-c and HDL-c. OR: odds ratio; CI: confidence interval.

## DISCUSSION

Coronary artery disease is the one of the leading causes of death, and is a serious public health problem worldwide. So far, the identified factors correlated with coronary artery disease were environment, ethnicity, family history and genetic mutation. Currently, many studies have shown that chronic inflammation could be a potential role in the development of cardiovascular diseases.^14^,^20^-^24^ The continuous condition of inflammation produces chronic damage promoting development and progression of certain types of cardiovascular diseases.^21^-^24^ In our study, we found that the *IL-6* gene polymorphisms is the risk factor of coronary artery disease susceptibility, and we observed that *IL-6* -592A>C polymorphism was correlated with higher risk of coronary artery disease in the Chinese population.

The IL-6 gene, located at chromosome 7p21-24, is composed of 4 introns and 5 exons. IL-6 could be considered as a major regulator of cardiovascular disease initiation and progression through circulating levels alternation. Al Shahi et al. carried out a study with 20 acute coronary syndrome patients and 50 stable coronary artery disease patients, and discovered that the higher plasma levels of IL-6 was associated with the pathogenesis of atherosclerosis.^25^ Eiras et al. reported that epicardial adipose tissue of coronary artery diseases was associated with higher levels of IL-6 mRNA than that of non-coronary artery disease patients.^26^ Another study was carried out in The Netherlands, and reported that plasma levels of IL-6 was significant higher in coronary artery disease compared to healthy controls.^27^

Genetic polymorphisms can change the structure and quantity of the gene product, ultimately affecting the function of the product.^28^ The IL-6 genetic polymorphisms may influence the expression and function of IL-6 protein, and thus affect the susceptibility to cardiovascular diseases. Previous studies have investigated the association between IL-6 genetic polymorphisms and development of cardiovascular diseases, but the results are conflicting.^14^,^16^,^18^,^22^,^29^ Li et al. carried out a study in Chinese population, and discovered that the CC genotype of *IL-6*-174G/C and the GG genotype of*IL-6*-572C/G are correlated with elevated risk of coronary artery disease.^16^ Wang et al. carried out a study with 402 patients with coronary artery disease and 402 control individuals, and reported a significant association between *IL-6* -174G>C polymorphism and coronary artery disease susceptibility, whereas no association was observed between *IL-6*-572C/G and this disease.^18^

A Chinese study found a significant correlation between *IL-6* -174G>C genomic variation and onset of cardiovascular events in patients receiving hemodialysis, but no correlation was observed in *IL-6*-572C/G genomic variation and risk of this disease.^22^ Gigante et al. carried out a study in two large populations, and reported that IL-6 haplotypes could regulate the circulating levels of inflammatory biomarkers and affect the susceptibility to coronary artery disease.^29^ Buraczynska et al. reported that C allele of the *IL-6* -174G>C was correlated with a significantly higher risk of cardiovascular disease compared to the G allele.^14^ Our study indicated that a significant relationship between *IL-6*-572C/G genetic polymorphism and coronary artery disease susceptibility. The discrepancies of the above mentioned studies may be attributed to differences in populations, disease status of coronary artery disease or sample size.

### Limitations of the study

First, study subjects were selected from only one hospital, which may cause selection bias. However, the genotype frequencies of *IL-6*-174G>C, -592G>C and -597G>A confirmed with the HWE, which suggests the genotype frequencies has representative of the general population. Second, our study may overlook the possibility of gene-gene interactions. Third, the sample size of our study is relatively small, which may limit the statistical power to find differences between groups. Further investigations with more sample size are needed to confirm our findings.

## CONCLUSION

This study suggests that *IL-6* -592G>C polymorphism is correlated with the risk of coronary artery disease. More well-designed prospective study based on large sample size, multiple SNPs or haplotypes are required to confirm the current findings.
